# Chinese Yam and Its Active Components Regulate the Structure of Gut Microbiota and Indole-like Metabolites in Anaerobic Fermentation In Vitro

**DOI:** 10.3390/nu15245112

**Published:** 2023-12-14

**Authors:** Yifan Cui, Yingzhuo Zhou, Yan Li, Jieying Wang, Daotong Li, Fang Chen

**Affiliations:** 1 National Engineering Research Centre for Fruits and Vegetables Processing, Key Laboratory of Fruits and Vegetables Processing, College of Food Science and Nutritional Engineering, Ministry of Agriculture, Engineering Research Centre for Fruits and Vegetables Processing, Ministry of Education, China Agricultural University, Beijing 100083, China; cuiyifan022@163.com (Y.C.); 18321782631@163.com (Y.Z.); ly13770618548@163.com (Y.L.); wangjieying@cau.edu.cn (J.W.); lidaotong@bjmu.edu.cn (D.L.); 2 Nutritional Biology, Division of Human Nutrition, Wageningen University & Research, 6708 WE Wageningen, The Netherlands

**Keywords:** Chinese yam, diosgenin, in vitro fermentation, gut microbiota, indole-like metabolites

## Abstract

As a medicinal and edible plant, Chinese yam (CY) can promote the enrichment of intestinal probiotics. Mucilage polysaccharides, diosgenin and taxifolin are the dominant components of CY. The purpose of this study was to investigate whether the impact of Chinese yam on gut microbiome structure and metabolism is attributable to its components. In the in vitro gastrointestinal digestion and colon fermentation system, the changes in gut microbiota composition and function were determined by 16S rRNA sequencing, and the levels of bacterial metabolites including short-chain fatty acids (SCFAs) and indole-like metabolites were detected by gas chromatography and an enzyme-linked immunoassay. The results show that CY, mucilage polysaccharides, diosgenin and taxifolin could increase the microbial diversity index. Furthermore, probiotics including *Lactobacillus* and *Bacteroides* were significantly increased, while harmful bacteria such as *Escherichia* and *Proteus* declined. CY could increase the production of SCFAs including acetic acid and butyric acid. Of note, CY and diosgenin displayed similar impacts on enhancing the abundance of *Clostridium* and promoting the production of indole-3-lactic acid and lactic acid. These findings provide evidence supporting Chinese yam as a natural food to regulate intestinal health. Diosgenin as a component of CY contributes mostly to the impact on regulating intestinal flora.

## 1. Introduction

The gut microbiome is increasingly recognized as a primary organ with involvement in nutrient metabolism and absorption [1]. The bacteria colonizing the digestive tract of animals can be beneficial or harmful [2]. The beneficial bacteria, including *Bifidobacterium*, *Bacteroidales* and *Akkermansia muciniphila*, play a vital role in maintaining gut homeostasis [3,4]. It is also reported that these beneficial bacteria can prioritize the use of fermenting non-digestible carbohydrates to produce bioactive metabolites such as short-chain fatty acids (SCFAs), which can be used by the host as precursors for the biosynthesis of lipids and proteins or as an energy source [5,6,7,8]. In addition to SCFAs, other gut microbial metabolites related to amino acid metabolism and carbohydrate fermentation, such as indole-like metabolites, are attracting more and more attention [9,10]. For instance, *Lactobacillus* and *Clostridium butyricum* can convert dietary tryptophan into indole-acetic acid (IAA) and indole-3-lactic acid (ILA), which have been implicated in the regulation of lymphocyte function [11,12].

Chinese yam, scientifically known as *Dioscorea opposita* Thunb., has a long history of cultivation spanning over three thousand years in East Asia [13]. It serves both as a staple food crop and a widely utilized ingredient in traditional Chinese medicine [14,15]. Studies have revealed the nutritional and medicinal properties of CY; for example, it effectively activated macrophages in vitro and promoted the production of NO, IL-6 and TNF-a [16]. Its components can trigger tumor necrosis, apoptosis and activation of antitumor immunity in C57BL/6 mice [17]. Remarkably, CY water decoction has exhibited efficacy in enhancing gastrointestinal peristalsis and restoring the metabolic phenotype, thus ameliorating intestinal disorders [18]. However, there are relatively few studies on the effects of CY on gut microbiota.

CY boasts an abundance of valuable nutrients, including resistant starches, steroidal sapogenins like diosgenin, mucilage polysaccharides and flavonoids such as taxifolin [19]. Chinese yam mucilage polysaccharides (CYPs) have been found to impede gastric emptying and small intestinal propulsion while facilitating intestinal absorption in mice with spleen deficiency [20]. CYPs were found to modulate the gut microbiota by reducing the levels of *Desulfovibrio* and *Sutterella* while concurrently elevating the abundance of *Prevotella* [21]. Diosgenin, which is recognized as a significant active substance in CY, exhibits gastric-ulcer-improving properties and boosts immunity by regulating intestinal flora *in vivo*, thereby safeguarding gastric mucosa cells and fortifying the gastrointestinal mucosa against harmful substances [22]. Taxifolin, as a bioflavonoid found abundantly in Tiegun yam, possesses potent anti-inflammatory and neuroprotective effects [23]. It also improved gut microbiota diversity and prevented *Proteobacteria* from blooming [24]. These results demonstrate that CY ingredients have a considerable influence on shaping the gut microbiota. Nevertheless, whether these components play a role in contributing to the impact of CY on gut microbiota remains unknown.

In vitro fermentative models are considered tools that allow the screening of substances to assess how they alter and are altered by gastrointestinal environments and microbial populations. They are widely applied to predict the influence of foods or nutrients on gut microbiota because these models are relatively inexpensive and without ethical concerns, which enables us to bind food, intake route and gut microbiota together [25]. In vitro batch fermentation models have recently been utilized for studying contribution of food to gut microbiota composition and functionality [26]. This methodology provides the investigator with the ability to access numerous substrate samples, either as actual food in its cooked or raw form or specific food components like dietary fibers or phenolic compounds [27]. It could help us to formulate initial hypotheses and facilitate the foundation of future trials.

In this study, we used an in vitro simulated digestion and fermentation model to investigate the effects of CY on the gut microbial function and metabolism. We further aimed to compare the differences between the effects of Chinese yam and its active components (CYPs, diosgenin, taxifolin) on intestinal flora function and indole-like compounds’ metabolism. Our results provide a scientific basis for future dietary interventions using CY to achieve intestinal homeostasis.

## 2. Materials and Methods

### 2.1. Materials

Diosgenin and taxifolin were purchased from Solarbio Science & Technology (Beijing, China). Trypsin (>99%) from porcine pancreas, pepsin (>99%), mucin from porcine stomach, L-cysteine (97%) and methanol (>99%) were purchased from Sigma Aldrich (St. Louis, MO, USA). Bile salt No. 3 (>99%), peptone and yeast extract were purchased from Aobox Biotechnology (Beijing, China). Twee-80 was purchased from Yuanye Bio-Technology (Shanghai, China). Chemicals studied in the article are listed in Appendix A.

### 2.2. Preparation of CY and CYPs

Fresh yams were supplied by Xincheng Huai Pharmaceutical Co., Ltd. (Jiaozuo, China). CY was peeled and steam-cooked and then cut into slices and freeze-dried for 48 h. The dried CY was pulverized with a blender (KA-2610, Jworld Tech, Ansan, Korea) for 30 s, screened through a 100-mesh sieve and stored at −20 °C until use. The proximate analysis of the CY powder was performed by the Agricultural Products Testing Center (Beijing, China).

CY slices mixed with distilled water (1:2, *w*/*v*) were homogenized at a speed of 10,000 r/min for 60 s. Subsequently, the mixture was left at room temperature for 3 h and centrifuged it at 4000 rpm for 5 min. The combined supernatant was concentrated and then mixed with absolute ethanol (1:4, *v*/*v*) at 4 °C for 12 h. The collected precipitate (4000 rpm, 5 min) was dissolved in distilled water and subjected to freeze drying, obtaining crude CYPs.

### 2.3. In Vitro Gastrointestinal Digestion of Chinese Yam and Its Components

Our procedure was based on previous methods with a few modifications [28]. The simulation of a system of gastric and small intestinal digestion with additions of Chinese yam and its components was achieved with an SPH-100D thermostat shaker (Shiping Instrument Corp., Shanghai, China). Firstly, 1 g of NaCl, 1.1 g of KCl, 0.15 g of CaCl_2_ and 0.6 g of NaHCO_3_ were dissolved in 1 L of distilled water to create the gastric medium. Then, 35.4 mg of gastric pepsin and 1.5 mL of CH_3_COONa (1.0 mol/L, PH = 5) were added into 100 mL of the medium, and the pH was adjusted to 2.0 by 0.1 M HCl solution to afford the simulated gastric fluid. The preparation of small intestinal fluid involved firstly dissolving 5.4 g of NaCl, 0.65 g of KCl and 0.33 g of CaCl_2_·2H_2_O in 1 L of distilled water. A 4% bile solution was prepared with Bile salt No. 3. Then, 14 g of pancreatic material was added into 200 mL distilled water to prepare pancreatic fluid. A combination of 50 mL of small intestinal fluid, 100 mL of bile solution, and 50 mL of pancreatic fluid was used to prepare 200 mL of small intestinal medium.

All digestions were performed under protection from light (by covering them with foil) and oxygen (using airtight containers). The pH of the gastric medium solution was maintained at 2.0. The pH of the small intestinal medium was maintained at 7.0.

### 2.4. Preparation of Fecal Flora Suspension

Fresh feces were collected from healthy, 8-week-old male C57BL/6J mice. The bacterial suspension from the supernatant was obtained after the feces and sterile PBS buffer (pH 7.0) were combined and diluted at a ratio of 1:9 (*w*/*v*), homogenized for 10 min and then centrifuged (3000 rpm, 5 min) to remove contaminants.

### 2.5. In Vitro Colonic Fermentation Model

The digestive fluids of yam extract, bacterial suspension and mixed medium were mixed at a volume ratio of 1:3:6. All samples were placed in the anaerobic-gas-generating bags from the Mitsubishi MGC AnaeroPackTM series, sealed and left in an anaerobic environment (20% CO_2_, 80% N_2_) and then incubated at a constant temperature of 37 °C. Fermentation was conducted for 24 h, and samples were collected at three time points (0, 12 and 24 h) for analysis. The fermentation broth was then centrifuged with a high-speed refrigerator centrifuge (12,000 rpm, 10 min), and the supernatant and sediment were collected and stored at −80 °C for later use.

### 2.6. Analysis of pH and SCFAs

The liquid was collected after 0, 12 and 24 h of fermentation for pH detection. The fermentation product was centrifuged at 10,000× *g* for 10 min, and then the supernatant was transferred to a 10 mL screw-cap tube, and the pH value was measured with a pH S-3B instrument (Lai Chi Instrument Co., Ltd., Shanghai, China).

After fermentation, the sample was mixed thoroughly with 800 μL of deionized H_2_O and 200 μL of 50% H_2_SO_4_. The mixture was added to 1 mL of diethyl ether and shaken by vortex for 10 s. The supernatant was collected after centrifugation (12,000× *g*, 10 min) and dehydrated by anhydrous CaCl_2_. The resulting supernatant was analyzed on a gas chromatograph using a hydrogen flame detector (flame ionization detector, FID) equipped with an SH-Stabil wax-DA (30 m × 0.32 mm × 0.50 μm). A flame ionization detector was used with an injector temperature of 260 °C, followed by temperature programming by holding the temperature at 80 °C for 1.5 min, increasing it to 240 °C at a rate of 10 °C/min and holding at 260 °C for 20 min. Nitrogen was used throughout the measurement process as a carrier gas. The concentrations of acetate, propionate, butyrate, isobutyrate, valerate and isovalerate were calculated based on the peak area of standard samples (Sigma Aldrich, St. Louis, MO, USA). The injection volume was 1 μL, and the injection was repeated 3 times.

### 2.7. Determination of Indole-like Compounds

The enzyme-linked immunosorbent assay (Meimian Industrial Co., Ltd., Yancheng, China) was used to determine lactic acid (LA), tryptophan (Trp), indole-3-lactic acid (ILA) and indoleacetic acid (IAA) in the gastrointestinal digestion and fermentation in vitro system at different times (0, 12 and 24 h). We measured the absorbance of each sample using a multimode microplate reader (TECAN, Männedorf, Switzerland) at a wavelength of 450 nm. The calculation methods for determining the content of indole compounds are listed in Supplementary Materials.

### 2.8. Processing and Analysis of 16S rRNA Gene Sequencing

Extracted total DNA from the fermentation fluid of CY and its active ingredients were analyzed using the TIANamp Stool DNA Kit (Tiangen, Beijing, China). Genomic DNA was utilized as a template for PCR amplification of the V3/V4 region of the bacterial 16S rRNA gene. PCR amplification products were analyzed with an RDP Classifier (http://rdp.cme.msu.edu/, accessed on 3 April 2023) against the Silva (SSU115) 16S rRNA database using a confidence threshold of 70%. Raw fastq files were demultiplexed and quality-filtered using QIIME (version 1.17) and further classified into operational taxonomic units (OTUs) within 0.03 (equivalent to 97% similarity) difference. Community richness was evaluated using Chao1, Sobs and Ace indexes. Principal coordinates analysis (PCoA) was applied to quantify the compositional differences between microbial communities. To identify bacterial taxa that were differentially represented between groups at the genus or higher taxonomic levels, linear discriminant analysis effect size (LEfSe) was applied to the present features followed by linear discriminant analysis (LDA) to measure the effect size of each abundant taxon and two filters (*p* < 0.05 and LDA score of >3.5). The heatmap was generated by the R package heatmap (R 4.2.1).

### 2.9. Statistical Analysis

Each experiment was repeated three times. All data are expressed as the mean ± SEM. Comparison of multiple samples was conducted by one-way analysis of variance (ANOVA) using SPSS software (version 25.0). Means with different letter superscripts indicate significant differences (*p* < 0.05). * *p* < 0.05 vs. 0 h, # *p* < 0.05 vs. 12 h in the tables. Plotting was performed with GraphPad Prism 7. Spearman’s rho nonparametric correlations among the gut microbes and indole compounds were then calculated, and the results were visualized with R (R 4.2.1).

## 3. Results

### 3.1. Establishment of In Vitro Digestion and Fermentation Models of CY and Its Components

**Figure 1:**
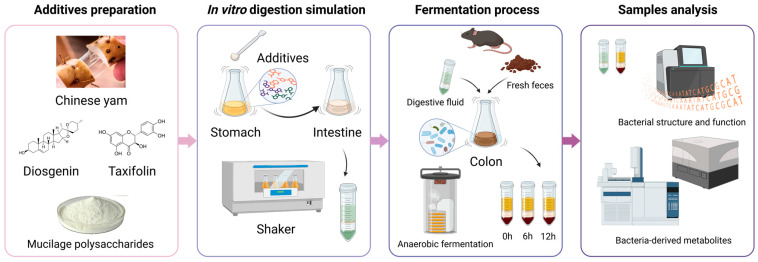
CY and its active ingredients in the in vitro simulation experimental process.

The in vitro simulation test of CY and its active ingredients comprised four stages, as depicted in Figure 1: additive preparation, digestion simulation, the fermentation process, and sample detection and analysis. Based on different additives, there were five groups, including the control, Chinese yam, CY mucilage polysaccharides, diosgenin and taxifolin. The CY amount considered was in accordance with the dietary recommendations for Chinese citizens. Meanwhile, the amounts of CYPs, diosgenin and taxifolin were calculated based on their content within CY. As shown in Table 1, the specific amounts utilized were 400 mg of CY, 139 mg of CYPs, 6.4 mg of diosgenin and 1.64 mg of taxifolin. Throughout the stomach and intestinal digestion simulation and colon anaerobic fermentation, the digestion and fermentation fluid was collected to further investigate the composition of the gut microbiota. Simultaneously, the levels of relevant metabolites were measured to assess bacterial metabolic function.

### 3.2. Improvement in Chinese Yam and Its Components in Terms of Bacterial Richness and Diversity after In Vitro Simulation Trial

**Figure 2:**
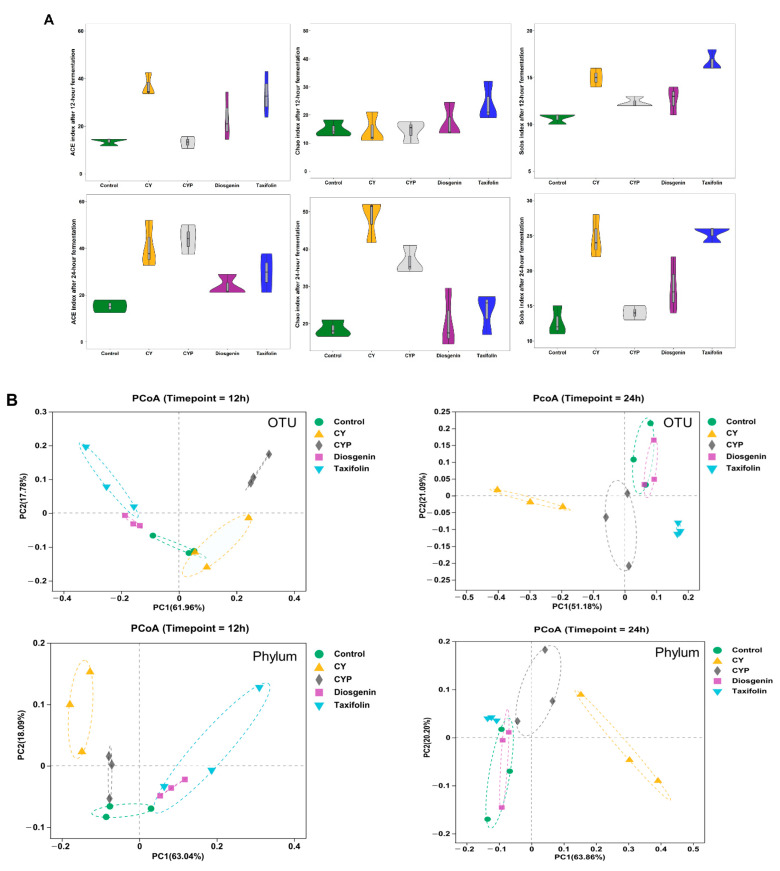

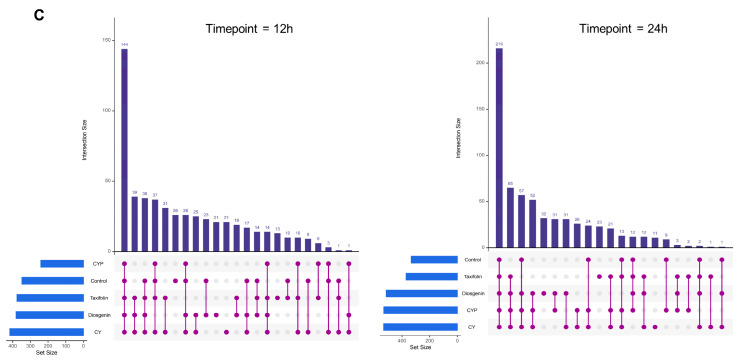
Effect of CY and its active ingredients on alpha and beta diversity of gut microbiota after in vitro fermentation. (**A**) Violin plots of ACE, Chao and Sobs index for different groups at the operational taxonomic unit (OTU) level at 12 h and 24 h. Wilcoxon rank-sum test; (**B**) weighted UniFrac principal coordinate analysis (PCoA) plots of the gut microbiota at the OTU and phylum levels. ANOSIM analysis; (**C**) UpSet plots among different groups based on OTUs at 12 h and 24 h.

α-diversity, a fundamental concept in microbial ecology, encompasses both the richness and proportion of gut microbiota. To assess the α-diversity, three common indexes (ACE, Chao and Sobs) were calculated. Violin plots show both the richness and diversity values of samples as well as their densities (Figure 2A). Compared with the fermentation liquid with Chinese yam components, the CY additive obviously enriched the abundance of the bacterial community, especially that of Sobs and ACE. The α-diversity of the fermentation mixture supplemented with CYPs and taxifolin moderately increased, whereas diosgenin did not have a notable impact on the abundance of gut microbiota. As the fermentation time increased, there was a concurrent rise in the abundance of the microbiota. The structure of gut microbiota in different fermentation systems was investigated by UniFrac-based PCoA. The results in Figure 2B show the bacterial composition at the OTU and phylum levels, exhibiting a distinct clustering pattern among the control, CY, CYP, diosgenin and taxifolin groups at different fermentation times. The CY group in particular was clearly distinguished from other in vitro treatments. In addition to Wayne diagrams, UpSet plots offer a clear visual summary of the intersections for the OTUs in multiple groups. These consist of a series of bars or line segments representing each subset, and the intersections are indicated by connected dots or lines. As shown in Figure 2C, the fermentation system of the CY group had the highest number of OTUs in comparison with the control and other groups. The number of OTUs was 417 at 12 h of fermentation, while the figure for 24 h of fermentation was 533. After the 12 h fermentation period, the number of intersections of OTUs among all groups was 144, while the figure for 24 h of fermentation was 215. After the 12 h fermentation period, the intersections of OTUs among the CY, CYP, diosgenin and taxifolin treatment groups numbered 14, while the figure for 24 h of fermentation was 65. At 12 h of fermentation, the number of intersections of OTUs in the CY group was 21, while the figure for 24 h of fermentation was 11. The number of intersections of OTUs among the CYP group, diosgenin group and taxifolin group was three for 24 h of fermentation, but there was not a shared figure for 12 h of fermentation. At 12 h of fermentation, the number of intersections of OTUs between the diosgenin group and taxifolin group was 19.

### 3.3. Effects of CY and Its Active Ingredients on the Composition and Structure of Microbiota In Vitro

**Figure 3:**
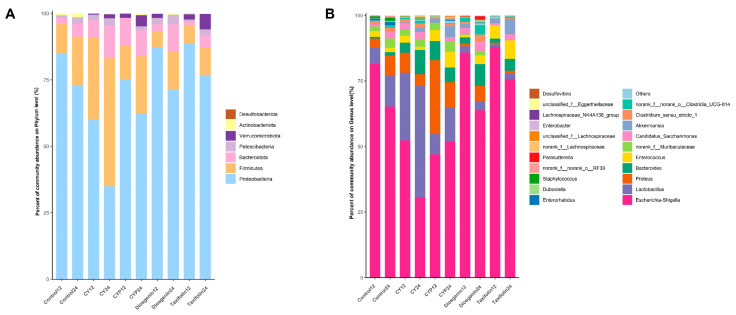
Relative abundance of bacterial community for each group after in vitro batch fermentation for 12 h and 24 h. (**A**) A bar graph of community composition at the phylum level; (**B**) a bar graph of community composition at the genus level.

To further investigate the control of bacteriophages during simulated in vitro fermentation of yam and its active components, high-throughput sequencing of the 16S rRNA gene was conducted at the phylum and genus levels. At the phylum level, the dominant bacterial populations in the broth samples at 12 h of fermentation were Proteobacteria, Firmicutes, Bacteroidota and Patescibacteria (Figure 3A). The abundance of Firmicutes obviously increased after the treatment of yam compared with the fermentation liquid with CY components. Both the CYPs and diosgenin moderately increased the abundance of Firmicutes and Bacteroidota. Moreover, taxifolin significantly enriched the level of Verrucomicrobiota. Compared with taxifolin and diosgenin treatment, CY showed a more obvious regulatory effect on the change in bacterial community structure after in vitro fermentation, which may be due to the richer nutrient content of the yam treatment.

There were distinct differences in the microbial composition among treatments at the genus level (Figure 3B). Compared with the control treatment after 12 h of fermentation, the abundance of *Lactobacillus* rose by roughly three times (*p* < 0.001) and that of *Bacteroides* by almost five-fold (*p* = 0.002) with the addition of CY, while CY decreased the abundance of *Escherichia–Shigella* by 35.8% (*p* < 0.001). The effect of the regulation of CYPs on the abundance of *Bacteroides* was extremely significant, increasing ten-fold (*p* < 0.001), and similarly reduced the abundance of *Escherichia–Shigella* by 42.27% (*p* < 0.001). When it comes to diosgenin and taxifolin, they both enhanced the amount of *Bacteroides* by, respectively, 273.54% (*p* = 0.042) and 140.42% (*p* = 0.26). Diosgenin specifically increased the abundance of *Akkermansia* (*p* = 0.009). The bar plots demonstrate the bacterial composition after 24 h of fermentation; CY further increased the abundance of *Lactobacillus* by 255.02% (*p* < 0.001) and *Bacteroides* by 650.03% (*p* < 0.001), while it significantly reduced the abundance of *Escherichia–Shigella* by 53.14% (*p* < 0.001) in comparison with the control. In addition to that, it should be emphasized that the influence of the other components on the level of bacterial genus followed the same trend as observed at 12 h. Notably, the in vitro system with the addition of diosgenin also showed an increase in the production of *Clostridium*, although the effect was not as pronounced as that observed with yam. These results suggest that CY and its ingredients could change the structure and composition of gut microbiota at both the phylum and genus levels.

**Figure 4:**
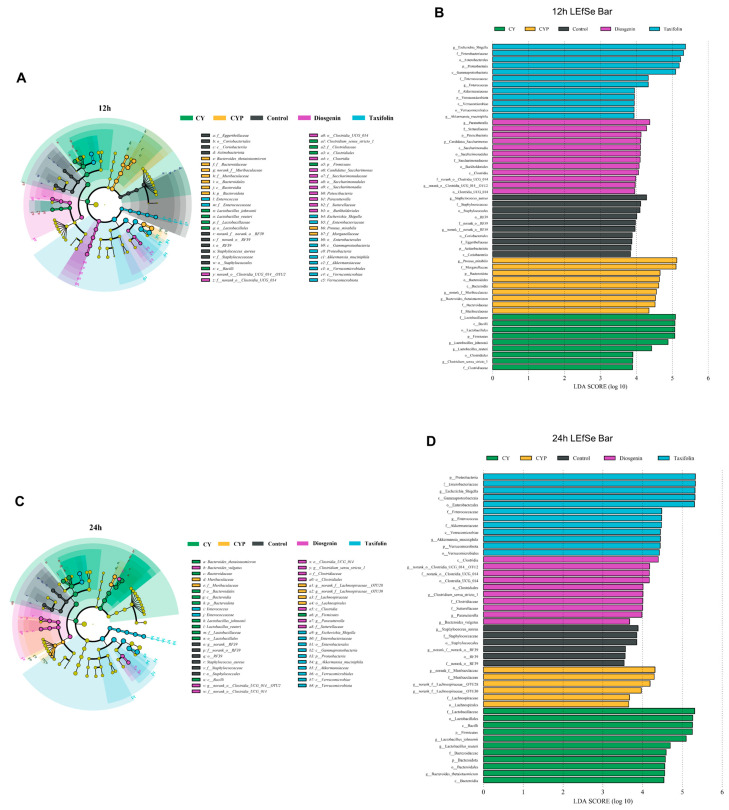
Effect of the CY and its active components on key microbial phylotypes, shown with a cladogram, and LDA scores derived from LEfSe analysis. (**A**,**B**) 12 h of fermentation and (**C**,**D**) 24 h of fermentation.

To further identify the effect of the regulation of CY on microbiota, LefSe was conducted to analyze the changes in microorganisms from the phylum to genus level. There were significant differences in the changes in microbiota by treatment group. LDA was applied to different species to distinguish between groups. After 12 h of fermentation (Figure 4A,B), *Lactobacillus_johnsonii*, *Lactobacillus_reuteri* and *Clostridium_sensu_stricto_1* were the most dominant genera of the CY group. *Proteus_mirabilis* and *Bacteroides_thetaiotaomicron* were the most dominant genera of the CYP group. *Parasutterella*, *Candidatus_Saccharimonas* and *Clostridia* were the most dominant genera under diosgenin treatment, while after the addition of taxifolin, both *Akkermansia_muciniphila* and *Enterococcus* were the dominant flora. Further, at the fermentation time point of 24 h (Figure 4C,D), the structure of the microbial community changed to some extent. The dominant flora with the addition of CY were still *Lactobacillus_johnsonii* and *Lactobacillus_reuteri*, whereas *Lachnospiraceae* was dominant in the CYP group, *Clostridium_sensu_stricto_1* and *Bacteroides_vulgatus* were dominant in the diosgenin group, and *Escherichia_Shigella*, *Enterococcus* and *Akkermansia_muciniphila* were dominant in the taxifolin group. It is proposed that CY and its active components can improve the microbiome structure in part of the colon in vitro, mainly through the abundance of *Lactobacillus*, *Akkermansia* and *Clostridium*.

### 3.4. Effects of CY and Its Active Components on pH and SCFA Production In Vitro

SCFAs, also known as volatile fatty acids, are composed of 1-6 carbon atoms [29]. The number of SCFAs produced by intestinal microbiota largely depends on the types of prebiotics and the composition of intestinal flora [30]. Table 2 presents the results for SCFAs in an in vitro simulated system with CY treatment, whose levels obviously increased compared with the control group. The acetic acid, propionic acid and butyric acid contents were significantly improved after 12 h of fermentation (*p* < 0.05), while the figures for isobutyric acid and isovaleric acid show no difference. At the time point of 24 h, butyric and isobutyric acids were continuously produced with the influence of CY, while the levels of other SCFAs did not change significantly in comparison with the control. The level of total SCFAs significantly increased by 57.9% in the control group and 69.2% in the CY group from the beginning stage to the intermediate stage of 12 h and declined at the 24 h fermentation stage. Overall, the addition of CY promoted the production of SCFAs in in vitro fermentation.

**Figure 5:**
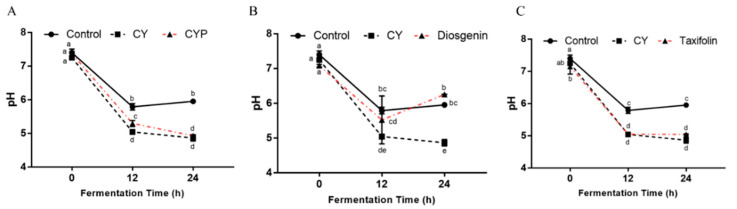
SCFAs and pH during in vitro fermentation. pH of control, CY and CYP (**A**) groups and diosgenin (**B**) and taxifolin (**C**) treatments at different time points during in vitro batch fermentation. Statistical analysis was performed by ANOVA (*p* < 0.05).

Changes in pH in simulated fermentation can provide insights into the microbial community’s function in organic acid metabolism. As shown in Figure 5A, compared with the control group, the pH of the in vitro simulated system after CY treatment was significantly reduced (*p* < 0.05). At 12 h of fermentation, the pH of the CYP and taxifolin groups (Figure 5B) was significantly lower than that of the control group and similar to that of the CY group (*p* < 0.05), and the same results were observed at 24 h. At the intermediate stage of fermentation, the pH of the diosgenin group (Figure 5C) was between that of the CY group and the control group, but increased at the end of fermentation and was significantly different from that of the in vitro simulated system after CY treatment (*p* < 0.05).

### 3.5. Effects of CY and Its Active Components on Specific Metabolites during In Vitro Digestive and Fermentation

IAA, as one of key tryptophan metabolites, can be produced by *Clostridium* [31]. As shown in Table 3, there was no significant difference in IAA content among all groups at the early stage of fermentation, while there was a noticeable rise in the fermentation system treated with CY in 24 h. With the extension of fermentation time, the concentration of IAA in different treatments, except the CYP group, was significantly increased (*p* < 0.05). The increased proportions were 14.8% in the control group, 7.67% in the CY group, 10.8% in the diosgenin group and 5.52% in the taxifolin group.

Furthermore, some bacteria belonging to the *Lactobacillus* genus have the ability to convert tryptophan into an intermediate known as ILA [32]. As shown in Table 4, during the fermentation process, there were no significant differences in ILA content among the five groups initially (*p* > 0.05). However, at the later stage of fermentation, the addition of CY to the samples significantly enhanced the ILA concentration compared with the control group (*p* < 0.05); meanwhile, an increasing trend in the diosgenin treatment group was also noticeable. However, the presence of other components in the fermentation samples did not lead to a significant difference in ILA content (*p* > 0.05). As the fermentation time progressed, the in vitro system supplemented with CY and taxifolin exhibited a significant increase in ILA content (*p* < 0.05). These findings, combined with the results of microbiological analysis, suggest that CY and its components may enhance ILA production by improving the abundance of *Lactobacillus*.

Lactic acid is an organic compound formed through the fermentation of carbohydrates. The primary bacteria responsible for lactic acid production are lactic acid bacteria, with certain strains of *Lactobacillus spp.* exhibiting particularly noteworthy fermentation capabilities [33]. During the in vitro fermentation process (Table 5), there was no significant difference in LA content in any group (*p* > 0.05). With the prolongation of fermentation time, the content of LA in CY and diosgenin fermentation solution significantly increased (*p* > 0.05). This result is related to the fact that CY and diosgenin can significantly increase the abundance of lactic acid bacteria.

Tryptophan is an essential amino acid which is obtained through diet. Certain bacteria, including *Lactobacillus* and *Clostridium*, can metabolize tryptophan and convert it into indole metabolites like IAA and ILA [34]. Therefore, a decrease in basal amino acid levels can reflect the situation and structure of intestinal flora. As shown in Table 6, originally, the concentration of tryptophan under CY treatment was significantly higher than that of the other components (*p* < 0.05), indicating CY contains a certain amount of dietary tryptophan. On the other hand, there was no significant difference between groups at 12 h and 24 h (*p* > 0.05). The concentration of tryptophan decreased over the fermentation process in all groups. Furthermore, it decreased significantly in the taxifolin group after 24 h of fermentation (*p* < 0.05).

Overall, in the in vitro batch fermentation system, CY and its active ingredients generally increased IAA, ILA and LA levels while decreasing tryptophan concentration, indicating that some of the tryptophan is converted into indole metabolites.

### 3.6. Correlation Analysis of Gut Microbiota at the Genus Level and Indole-like Metabolites In Vitro

**Figure 6:**
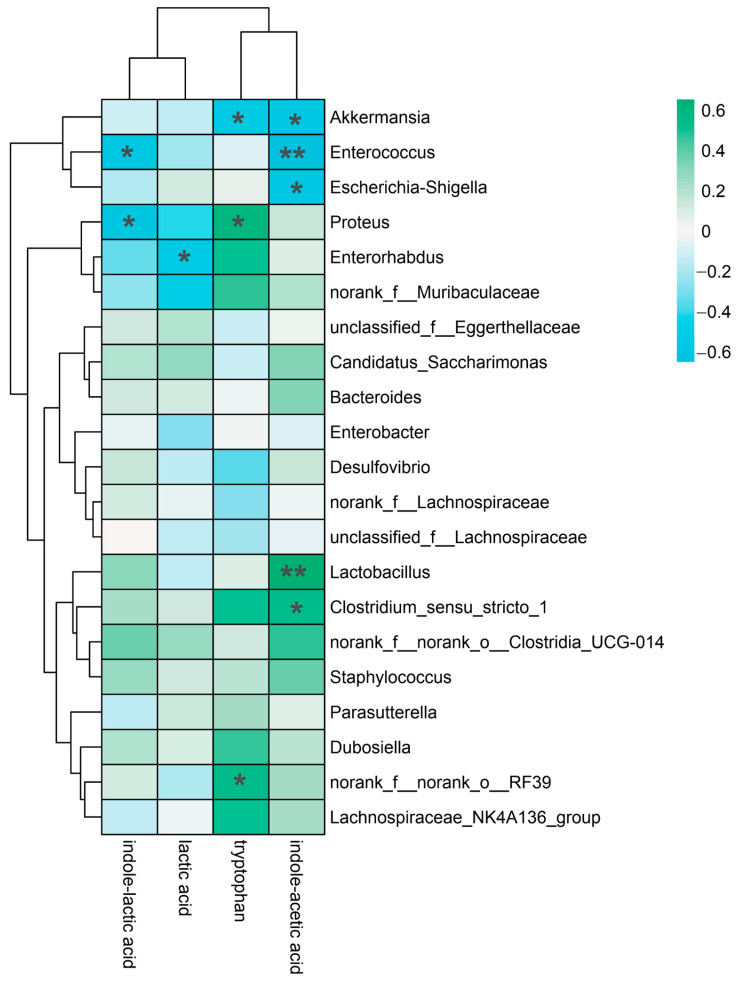
Spearman correlation analysis between the microbiota and indole-like metabolites. * *p* < 0.05, ** *p* < 0.01.

To distinguish the relationship between yam-modulated gut microbiota and metabolites, we performed a Spearman correlation analysis between gut microbiota and their metabolites (Figure 6). IAA demonstrated a highly substantial positive correlation with *Enterococcus* and *Lactobacillus* (*p* < 0.01) as well as a strong positive correlation with *Akkermansia*, *Escherichia coli* and *Clostridium* (*p* < 0.05). Tryptophan significantly and positively correlated with *Proteus*, *Akkermansia* and *norank_f__norank_o__RF39* (*p* < 0.05). LA and *Enterorhabdus* had a strong positive association (*p* < 0.05). ILA demonstrated a statistically significant positive connection with *Enterococci* and *Proteus* (*p* < 0.05). These correlation analyses indicated that the growth in microbiota contributed to the production of tryptophan metabolites.

## 4. Discussion

Research on the regulation of gut microbiota by medicinal and edible homologous plants is being supported by increasing evidence. Chinese yam, cultivated for medicinal and dietary applications, contains many bioactive components, including CYPs, diosgenin and dihydroquercetin [14]. It has been confirmed that CY possesses the capacity to induce targeted modifications in microbial communities and metabolic profiles [35]. In vitro digestion and colonic fermentation is regarded as an economic, convenient and reproducible method that can be used to study the effect of foods on intestinal flora and its metabolites compared with in vivo intervention trials. Researchers commonly use in vitro methods to obtain initial experimental insights and subsequently employ in vivo experiments to validate the findings [25]. At present, the specific active ingredients in CY that play a role in regulating the flora and the microbiota-derived metabolites remain unclear. The goal of our study was to examine the effects of CY and its active ingredients (CYPs, diosgenin, taxifolin) on the structure and functional metabolism of gut microbiota through anaerobic fermentation in vitro.

Initially, we observed that CY can boost the α-diversity of gut microbiota, indicating the significant effects of CY on microbiota diversity. Furthermore, the dominant bacterial populations in the broth samples at 12 h of fermentation were Proteobacteria, Firmicutes and Bacteroidota at the phylum level. After 24 h of fermentation, CY promoted the production of Firmicutes and Bacteroidota while decreasing the level of Proteobacteria. Moreover, CY could increase the abundance of *Lactobacillus* and *Akkermansia* while decreasing that of *Escherichia* and *Proteus* at the genus level. These findings suggest that CY can regulate the structure of gut microbiota. The potential prebiotic effect of CY on gut microbiota was mainly reflected in the promotion of the growth of *Lactobacillus* and *Akkermansia*. Lactic acid bacteria encompass a diverse group of microorganisms, and certain strains among them have been harnessed as probiotics due to their ability to metabolize tryptophan [36]. Similarly, an increased level of lactic acid bacteria was observed with the addition of CY peel in a freshwater aquaculture carp model [37]. Researchers have reported that *Lactobacillus* can regulate the balance of the host intestinal environment and improve the host immune protection ability and nutrition metabolism by promoting the transformation and absorption of nutrients [38].

SCFAs contribute to the acidification of the batch fermentation environment, which is consistent with the decrease in pH value. As expected, the pH values of the CY group showed a decreasing trend during the whole fermentation process, and the same trend could be observed in CYPs and taxifolin, indicating that CYPs and taxifolin could also function independently of yam. At the same time, the concentrations of acetic acid, propionic acid, butyric acid, isobutyric acid, isovaleric acid and total SCFAs were all higher in the supernatants from the fermentation broths with the digested CY, which is related to the basal nutrient medium utilization by the increased number of SCFA-producing bacteria caused by CY. In addition, the acidic colon environment can contribute to host health by promoting the proliferation of beneficial bacteria and inhibiting the growth of pathogens.

In recent years, there has been an increasing number of studies into the small-molecule metabolites produced by gut microbes, as they play a vital role in regulating the host’s pathophysiological state [39]. Previous research has documented alterations in the tryptophan metabolic profile when investigating the impact of yam intervention on antibiotic-induced rats [18]. Therefore, we also explored the changes in indole-like metabolites, such as IAA and ILA, over time during in vitro fermentation. IAA is one of the metabolites of tryptophan produced from *Clostridium* [31]. Tryptophan can also be metabolized by gut bacteria like *Lactobacillus reuteri* (*L. reuteri*) and *Lactobacillus murinus* (*L. murinus*) into ILA [40]. These indole-like compounds belong to tryptophan metabolites as well as the organic acid LA, and these indexes can be used to evaluate the potential prebiotic effect of CY. In this study, we observed that CY could promote an increase in IAA, ILA and LA concentrations in colonic fermentation, while reducing the concentration of tryptophan, which indicates that some tryptophan is converted into indole metabolites. The mediated influence of CY on promoting the production of tryptophan metabolites was very obvious, and its components were a partial effect. Nevertheless, the concentration of indole metabolites was positively correlated with the number of beneficial intestinal bacteria. These results are preliminary, and further in vivo studies are needed to determine whether these potential prebiotic effects have health benefits for humans.

In summary, this study revealed that CY could increase the output of SCFAs in the intestine, create an acidic intestinal environment and increase the richness of the flora. CY and its active components, like diosgenin and taxifolin, promoted the growth of beneficial bacteria such as *Lactobacillus*, *Clostridium* and *Akkermansia*. Both CY and diosgenin could promote the production of indole metabolites, which were positively correlated with the production of beneficial bacteria like *Lactobacillus* and *Clostridium* during in vitro fermentation. However, further research is needed to understand the underlying mechanisms by which the gut microbiota mediate yam metabolite production. Collectively, these findings highlight the potential importance of understanding the interaction between active ingredients of CY and gut microbiota in the context of host health.

## 5. Conclusions

In conclusion, during simulated gastrointestinal digestion and colon fermentation, CY and its active components were utilized by the gut microbiota, obviously improving microflora diversity at the phylum and OTU levels. CY, CYPs, diosgenin and taxifolin exerted a regulatory influence on the gut microbial composition compared with the control treatment. At the genus level, the relative abundance of beneficial bacteria like *Lactobacillus*, *Clostridium* and *Bacteroides* was enhanced. Concurrently, the amount of *Escherichia* and *Proteus* diminished. The addition of yam caused a significant increase in total SCFA levels, including acetic and butyric acids. Furthermore, CY and diosgenin demonstrated the capacity to stimulate the production of indole derivatives (ILA, IAA). The correlation analysis supported significant associations between specific metabolites of CY and related intestinal bacteria.

## Figures and Tables

**Table 1:** Additives of CY and its active components in in vitro anaerobic fermentation.

Additives	Content in CY (Dry Basis, %)	Amount (mg)
CY	100	400
CYPs	12.54	139
Diosgenin	0.9	6.4
Taxifolin	0.41	1.64

**Table 2:** The concentration of SCFAs between control and CY groups (μg/mL).

SCFA	Treatment	Fermentation Time (h)		
		0	12	24
Acetic acid	Control	65.62 ± 4.06 ^a^	115.74 ± 7.55 ^b^	77.15 ± 3.85 ^a^
	CY	82.88 ± 4.46 ^a^	145.75 ± 10.52 ^c^	123.69 ± 4.66 ^b^
Propionic acid	Control	12.44 ± 1.27 ^a^	15.05 ± 0.48 ^ab^	13.68 ± 0.49 ^a^
	CY	12.25 ± 0.33 ^a^	18.03 ± 0.75 ^c^	17.04 ± 1.47 ^bc^
Butyric acid	Control	10.24 ± 0.27 ^a^	13.95 ± 0.65 ^b^	14.41 ± 1.30 ^bc^
	CY	9.81 ± 0.34 ^a^	17.51 ± 1.31 ^c^	26.55 ± 1.69 ^d^
Iso-butyric acid	Control	1.73 ± 0.09 ^a^	3.98 ± 0.42 ^b^	4.13 ± 0.48 ^b^
	CY	1.59 ± 0.13 ^a^	4.72 ± 0.70 ^b^	3.75 ± 0.36 ^b^
Valeric acid	Control	6.45 ± 0.27 ^c^	6.18 ± 0.32 ^bc^	5.11 ± 0.13 ^a^
	CY	6.35 ± 0.17 ^c^	7.34 ± 0.14 ^d^	5.38 ± 0.48 ^ab^
Iso-valeric acid	Control	6.18 ± 0.31 ^ab^	7.21 ± 0.67 ^bc^	6.29 ± 0.19 ^ab^
	CY	6.27 ± 0.41 ^ab^	8.28 ± 0.63 ^c^	5.62 ± 0.30 ^a^
Total SCFA	Control	102.66 ± 5.30 ^a^	162.10 ± 8.11 ^b^	120.77 ± 3.79 ^ab^
	CY	119.15 ± 4.44 ^a^	201.63 ± 11.87 ^c^	182.02 ± 5.69 ^bc^

Means with different letter superscripts are considered to be significantly different (*p* < 0.05).

**Table 3:** The concentration of IAA at different fermentation times (ng/L).

Treatment	Fermentation Time (h)		
	0	12	24
Control	894.84 ± 54.89 ^a^	1051.73 ± 9.10 ^d^*	1027.06 ± 4.11 ^bc^*^#^
CY	978.08 ± 17.14 ^a^	1018.75 ± 8.87 ^c^	1053.33 ± 7.13 ^c^*^#^
CYPs	974.50 ± 42.88 ^a^	995.75 ± 9.22 ^bc^	989.96 ± 21.89 ^ab^
Diosgenin	926.07 ± 30.39 ^a^	981.19 ± 10.29 ^b^	1026.71 ± 16.33 ^bc^*^#^
Taxifolin	906.03 ± 16.18 ^a^	932.96 ± 3.00 ^a^*	956.01 ± 3.51 ^a^*

Means with different letter superscripts indicate significant difference (*p* < 0.05). * *p*< 0.05 vs. 0 h, # *p*< 0.05 vs. 12 h.

**Table 4:** The concentration of ILA at different fermentation times (ng/L).

Treatment	Fermentation Time (h)		
	0	12	24
Control	177.75 ± 11.77 ^a^	209.06 ± 6.19 ^a^	241.09 ± 11.42 ^a^*
CY	199.28 ± 12.12 ^a^	212.75 ± 17.16 ^a^	324.9 ± 23.38 ^b^*^#^
CYPs	162.34 ± 14.73 ^a^	173.37 ± 8.8 ^a^	176.39 ± 11.27 ^a^
Diosgenin	154.44 ± 18.69 ^a^	183.58 ± 18.14 ^a^	208.64 ± 29.98 ^a^
Taxifolin	163.79 ± 9.98 ^a^	173.72 ± 10.25 ^a^	236.65 ± 13.83 ^a^*^#^

Means with different letter superscripts indicate significant difference (*p* < 0.05). * *p* < 0.05 vs. 0 h, # *p* < 0.05 vs. 12 h.

**Table 5:** The concentration of LA at different fermentation times (μg/L).

Treatment	Fermentation Time (h)		
	0	12	24
Control	21.46 ± 0.62 ^a^	21.00 ± 0.31 ^bc^	21.80 ± 1.07 ^ab^
CY	20.26 ± 0.96 ^a^	20.05 ± 0.63 ^ab^	23.09 ± 0.14 ^b^*^#^
CYPs	20.88 ± 1.13 ^a^	19.19 ± 0.41 ^a^	20.50 ± 0.29 ^a^
Diosgenin	22.09 ± 0.64 ^a^	21.20 ± 0.11 ^bc^	22.95 ± 0.18 ^b#^
Taxifolin	21.77 ± 0.48 ^a^	22.22 ± 0.72 ^c^	22.77 ± 0.73 ^b^

Means with different letter superscripts indicate significant difference (*p* < 0.05). * *p* < 0.05 vs. 0 h, # *p* < 0.05 vs. 12 h.

**Table 6:** The concentration of tryptophan at different fermentation times (μmol/L).

Treatment	Fermentation Time (h)		
	0	12	24
Control	98.69 ± 0.38 ^ab^	98.06 ± 3.96 ^a^	95.09 ± 1.55 ^a^
CY	101.86 ± 1.62 ^b^	85.70 ± 7.36 ^a^	86.25 ± 5.25 ^a^
CYPs	95.31 ± 1.63 ^a^	93.13 ± 1.86 ^a^	93.09 ± 1.70 ^a^
Diosgenin	95.31 ± 1.37 ^a^	96.43 ± 0.19 ^a^	93.67 ± 0.25 ^a^
Taxifolin	94.93 ± 2.03 ^a^	91.29± 0.56 ^a^	88.48 ± 1.28 ^a^*

Means with different letter superscripts indicate significant difference (*p* < 0.05). * *p* < 0.05 vs. 0 h.

## Data Availability

Data are contained within the article.

## Supplementary Materials

The following supporting information can be downloaded at: https://www.mdpi.com/article/10.3390/nu15245112/s1, Figure S1: Calculation of the content of indole derivatives.

## Appendix A

Chemicals studied in the article: taxifolin (PubChem CID: 712316); diosgenin (PubChem CID: 99474); acetic acid (PubChem CID: 176); propionic acid (PubChem CID: 1032); butyric acid (PubChem CID: 264); iso-butyric acid (PubChem CID: 6590); valeric acid (PubChem CID: 7991); iso-valeric acid (PubChem CID: 10430); indole-acetic acid (PubChem CID: 802); indole-3-lactic acid (PubChem CID: 92904); lactic acid (PubChem CID: 612); tryptophan (PubChem CID: 6305).
