# Supplementary material for: Chinese Yam and Its Active Components Regulate the Structure of Gut Microbiota and Indole-like Metabolites in Anaerobic Fermentation In Vitro
Source: Nutrients. 2023 Dec 14;15(24):5112. doi: 10.3390/nu15245112 (PMC10746045; doi:10.3390/nu15245112)
Supplement: Supplementary file 1 [file nutrients-15-05112-s001.zip › nutrients-2723337-supplementary.pdf]

*Calculation of the content of indole derivatives*

Generate the standard curve using the concentration of the standard substance on the x-axis and the OD value on the y-axis. By substituting the OD value of the sample into the equation, determine the actual concentration of the sample.

The regression equation for the standard curve：

IAA： $y = - 0.0084x + 2.1965$; $R^2 = 0.996$.

LA： $y = - 0.003x + 1.8176$; $R^2=0.999$.

TRP： $y = -0.137x + 3.0972$; $R^2=0.985$.

ILA： $y = 9.3903x - 0.769$; $R^2=0.992$.
